# Supplementary material for: Neurological manifestations and MMP8 as a prognostic biomarker in severe fever with thrombocytopenia syndrome
Source: PLoS Negl Trop Dis. 2025 Dec 26;19(12):e0013875. doi: 10.1371/journal.pntd.0013875 (PMC12758824; doi:10.1371/journal.pntd.0013875)
Supplement: S3 Table — (DOCX) [file pntd.0013875.s003.docx]

**Table S3. Risk factors associated with 30-day mortality of SFTS patients by univariate and multivariate cox regression analysis**

|  | **Univariate analysis** | **P**  **value** | **Multivariate analysis** | **P**  **value** |
| --- | --- | --- | --- | --- |
|  | **HR (95% CI)** |  | **Adjusted HR (95% CI)** |  |
| Age (years) | 1.082(1.014-1.155) | 0.017 | 0.800(0.072-8.934) | 0.856 |
| Gender (Male) | 0.305(0.059-1.574) | 0.156 |  |  |
| Hypertension | 3.181(0.711-8.063) | 0.130 |  |  |
| Diabetes | 0.971(0117-8.063) | 0.978 |  |  |
| Tumor | 3.663(0.705-19.023) | 0.122 |  |  |
| WBC (×10^9^/ L) | 1.102(0.915-1.328) | 0.305 |  |  |
| RBC (×10^12^/L) | 1.445(0.438-4.766) | 0.546 |  |  |
| PLT (×10^9^/ L) | 0.966(0.933-1.001) | 0.054 | 0.965(0.319-2.923) | 0.950 |
| HB (g/L) | 1.025(0.983-1.069) | 0.246 |  |  |
| ALT (U/L) | 1.006(1.000-1.013) | 0.063 | 1.045(0.755-1.447) | 0.791 |
| AST (U/L) | 1.002(1.001-1.003) | <0.001 | 0.980(0.910-1.056) | 0.594 |
| LDH (U/L) | 1.001(1.000-1.001) | 0.001 | 0.983(0.919-1.051) | 0.610 |
| CK (U/L) | 1.000(1.000-1.000) | 0.065 | 1.001(1.000-1.001) | 0.043 |
| Cr (μmol/L) | 1.038(1.018-1.058) | <0.001 | 1.041(1.009-1.075) | 0.013 |
| BUN (mmol/L) | 1.358(1.156-1.597) | <0.001 | 2.159(0.002-1872.497) | 0.824 |
| CRP (mg/L) | 1.026(0.991-1.063) | 0.146 |  |  |
| D-dimer (ng/mL) | 1.241(1.095-1.406) | <0.001 | 1.257(1.069-1.478) | 0.006 |
| PT (s) | 1.219(0.999-1.488) | 0.051 | 1.185(0.000-44784.851) | 0.985 |
| APTT (s) | 1.078(1.036-1.122) | <0.001 | 1.935(0.157-23.913) | 0.607 |
| *MMP8* (ng/mL) | 1.026(1.004-1.047) | 0.018 | 1.060(1.010-1.112) | 0.018 |
| SFTSV RNA  (TCID50/ml) | 1.000(1.000-1.000) | 0.051 | 1.000(1.000-1.000) | 0.072 |

Risk factors were selected by univariate and multivariate cox regression analysis, especially, multivariate cox regression analysis was conducted by a forward stepwise method with predictor entry P < 0.10. Abbreviation: WBC, white blood cell; RBC, red blood cell; PLT, platelet; HB, hemoglobin; ALT, alanine aminotransferase; AST, aspartate aminotransferase; LDH, lactate dehydrogenase; CK, creatinine kinase; Cr, creatinine; BUN, blood urea nitrogen; CRP, C-reactive protein; PT, prothrombin time;

APTT, activated partial thromboplastin time; CI, confidence interval; HR, hazard ratio; PLT, platelet;
