# Supplementary figures and images for: Neurological manifestations and MMP8 as a prognostic biomarker in severe fever with thrombocytopenia syndrome
Source: PLoS Negl Trop Dis. 2025 Dec 26;19(12):e0013875. doi: 10.1371/journal.pntd.0013875 (PMC12758824; doi:10.1371/journal.pntd.0013875)

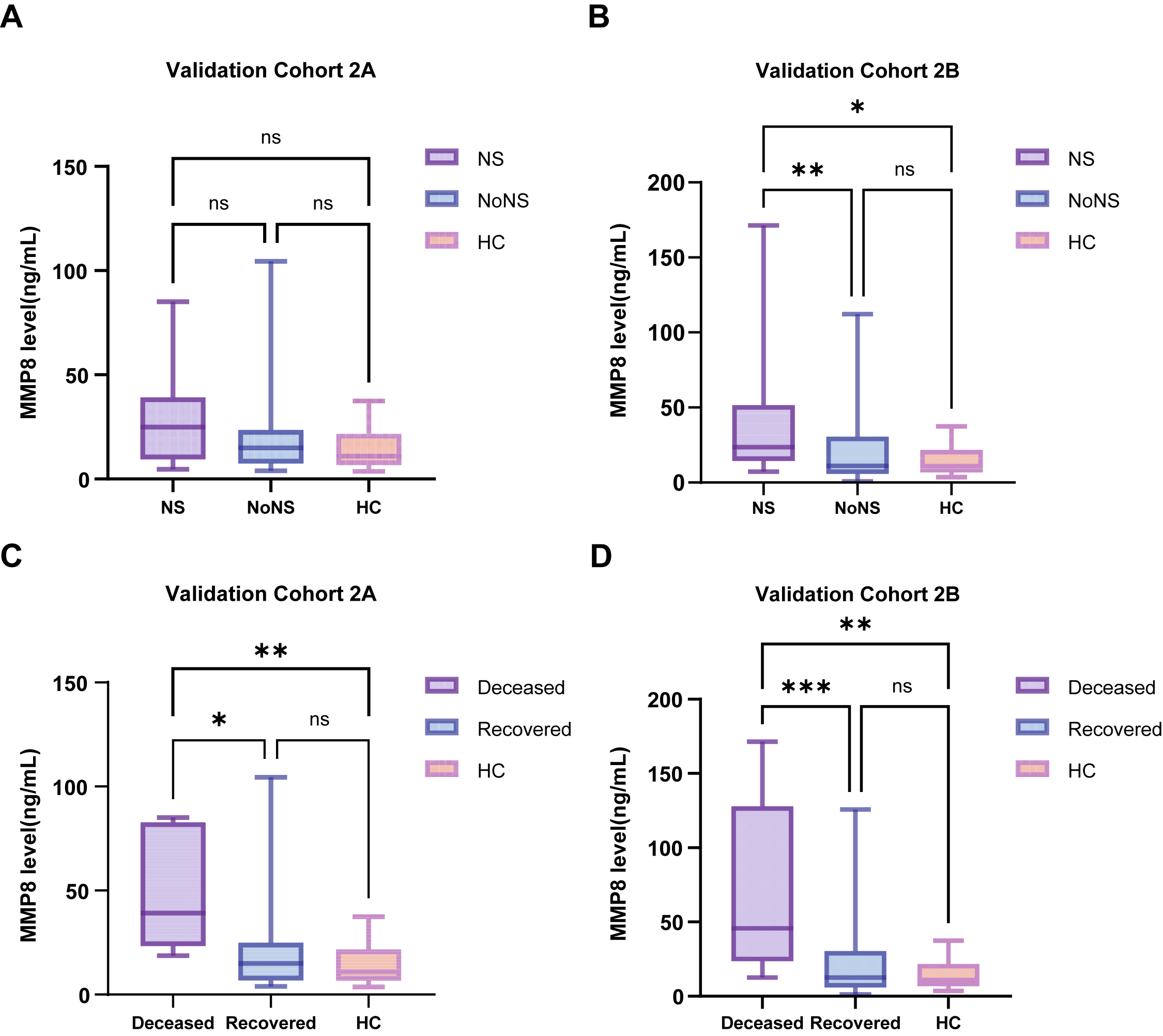

Supplement: S2 Fig — (A-B) Comparison of serum MMP8 concentrations among SFTS patients with neurological symptoms (NS), without neurological symptoms (noNS), and healthy controls in Validation Cohort 2A (A) and Cohort 2B (B). (C-D) Comparison of serum MMP8 levels among deceased patients, recovered patients, and healthy controls in Validation Cohort 2A (C) and Cohort 2B (D). Boxplots show the median, interquartile range, and full data range. Statistical significance across the three groups in each comparison was determined using the Kruskal-Wallis test with Dunn’s multiple comparisons test. Significant differences are indicated (*P < 0.05, **P < 0.01, ***P < 0.001, ****P < 0.0001). (TIF) [file pntd.0013875.s005.tif]

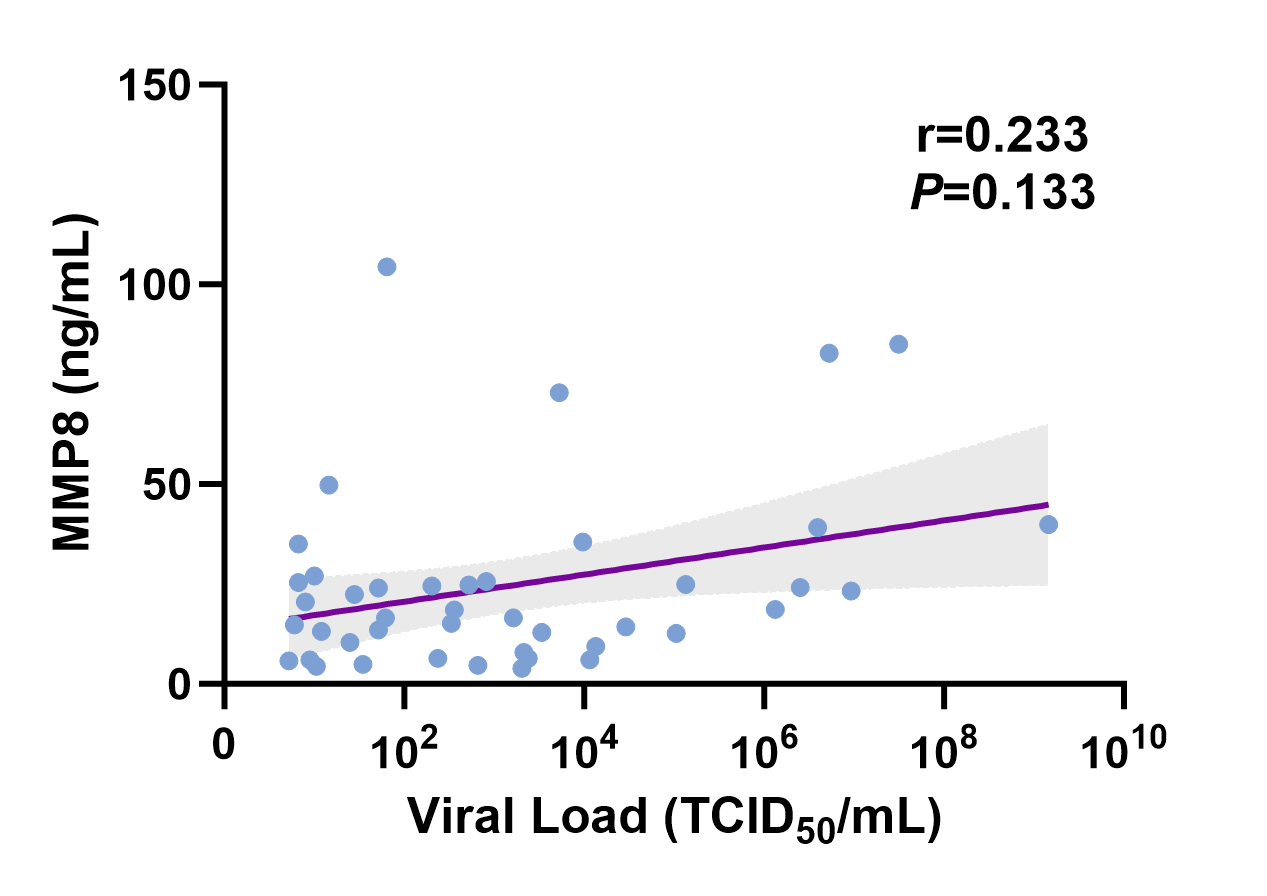

Supplement: S4 Fig — No significant correlation was observed between serum MMP8 (Y-axis) and viral load (X-axis) in SFTS patients (Spearman’s correlation, P = 0.133). (TIF) [file pntd.0013875.s007.tif]

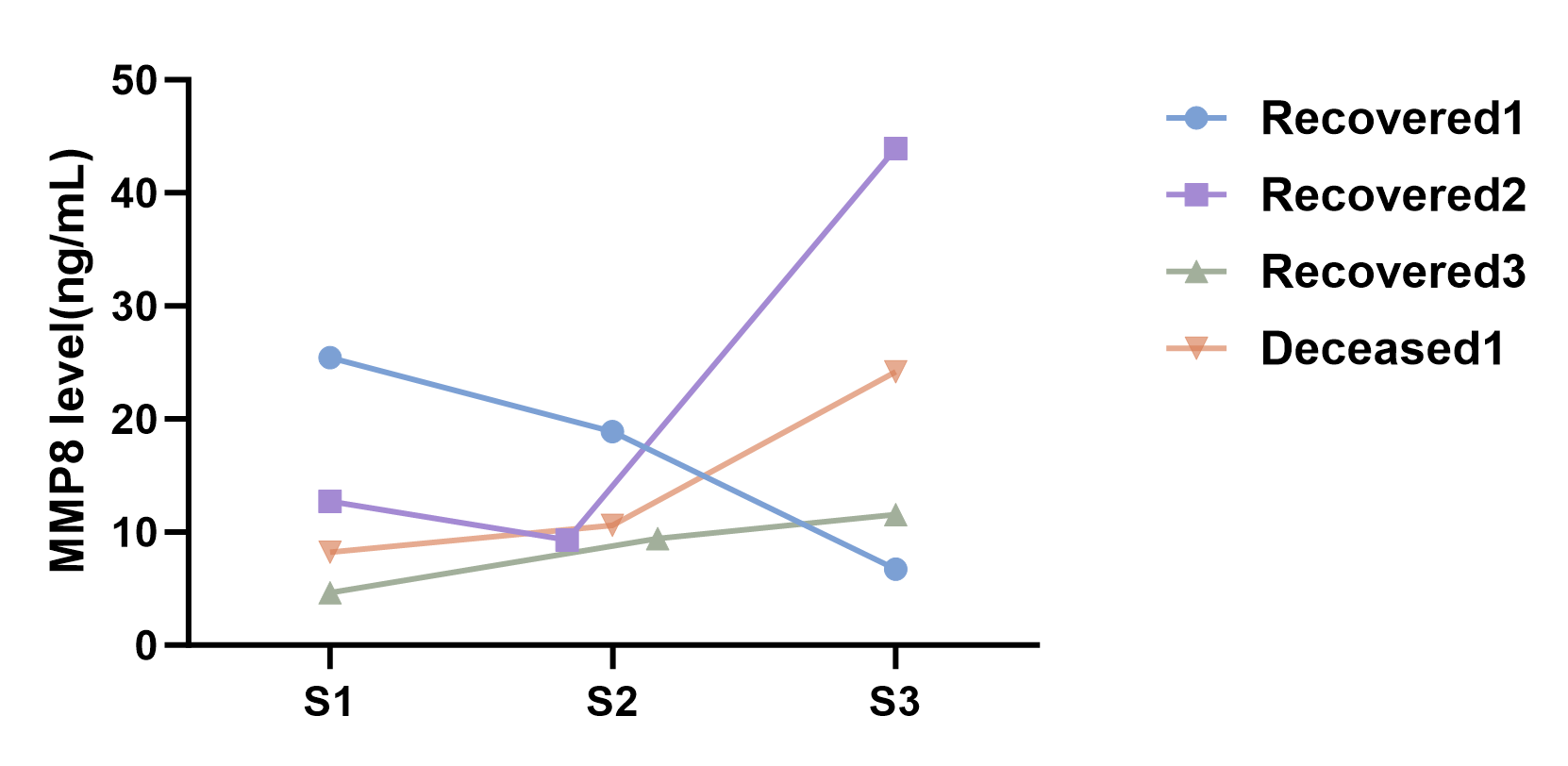

Supplement: S5 Fig — The line graph depicts the serum MMP8 concentration trajectories across different disease stages (S1, S2, S3) for four patients: one who ultimately died and three who recovered. A clear distinction in the patterns emerged: the fatal case showed a progressively rising trajectory, whereas the recovered patients displayed variable trends. This analysis is presented as preliminary and descriptive due to the small sample size; no formal statistical tests were performed. (TIF) [file pntd.0013875.s008.tif]
