# Supplementary material for: Neurological manifestations and MMP8 as a prognostic biomarker in severe fever with thrombocytopenia syndrome
Source: PLoS Negl Trop Dis. 2025 Dec 26;19(12):e0013875. doi: 10.1371/journal.pntd.0013875 (PMC12758824; doi:10.1371/journal.pntd.0013875)
Supplement: Attachment — Submitted filename: Response to reviewers.docx [file pntd.0013875.s009.docx]

**Point-by-Point Response to Reviewers' Comments**

**Review 1**

**Comment 1**: The study sample size is too small, and the comparisons and statistical analyses between groups lack persuasiveness. The sequencing analysis struggles to accurately demonstrate the pathogenesis. Simply highlighting a few genes with significant differences does not adequately reflect value at both the pathway and biomarker levels.

**Response:** We acknowledge the reviewers' concern regarding the limited sample size in the initial study. In the initial study design, although FOXJ1 and RGS4 identified by us showed associations with the disease at the transcriptome level, the lack of commercial serum ELISA detection kits for these two molecules made it difficult to conduct large-scale validation using peripheral blood samples, which are clinically easily accessible. This is particularly crucial as reviewers have repeatedly emphasized the significance of serum level validation for the clinical utility of a biomarker. In the revised manuscript, we have reanalyzed the original transcriptomic sequencing data by regrouping the samples according to the presence or absence of neurological symptoms. Based on this reanalysis, we have shifted our focus from FOXJ1 and RGS4 to MMP8, which emerged as a more promising candidate. Moreover, we have significantly expanded our validation efforts by collecting serum samples from 151 hospitalized SFTS patients across two independent centers (43 from Zhejiang University First Affiliated Hospital and 108 from Nanjing Drum Tower Hospital). This larger cohort was used to validate the prognostic value of MMP8, a key gene identified in our RNA-seq analysis. The serum MMP8 levels were measured via ELISA, and the results demonstrated robust discriminatory performance for predicting mortality (AUC = 0.821, 95% CI: 0.681–0.962 in the first cohort and AUC = 0.827, 95% CI: 0.721–0.932 in the second cohort). These findings were further supported by multivariate Cox regression analysis, which confirmed MMP8 as an independent predictor of mortality (HR: 1.527, 95% CI: 1.300–1.810). Additionally, we have added volcano plot of differentially expressed genes in the transcriptome of SFTS patients with and without neurological symptoms (Fig 4), as well as specific expression scales for upregulated genes (Table S2).

We have also refined our statistical methods, ensuring that all comparisons now meet the reviewers' standards for rigor. For example, we applied Mann-Whitney U tests and Cox regression models to strengthen the validity of our conclusions. We believe these additions substantially enhance the reliability and generalizability of our findings.

**Comment 2:** There is already a substantial amount of research on the screening of prognostic factors related to SFTS, even the construction of prognostic models, and the association between neurological symptoms and fatal outcomes is a widely acknowledged phenomenon. The study's simple statistical comparison of neurological symptoms with death lacks innovation.

**Response:** Thanks for the reviews comment. **While prior studies have suggested an association between neurological symptoms and mortality in SFTS, the specific neurological symptoms involved remained indistinct. We addressed this gap by subdividing neurological symptoms into discrete categories: consciousness disorder, headache, restlessness, tremor, convulsion, and mental state change. We aim to identify specific neurological symptoms which exhibit a strong association with mortality, thereby establishing a foundation for enhanced early prognostic assessment in clinical practice. The results demonstrated that consciousness disorder (p<0.001) and convulsions (p=0.003) were significant predictors of mortality.** Moreover, our study introduces a novel mechanistic insight by linking neurological symptoms to peripheral biomarkers (MMP8) with a potential biological rationale. Unlike prior studies that focused on clinical symptomatology alone, we provide a mechanism-driven approach by demonstrating that elevated serum MMP8 levels are strongly associated with fatal outcomes (AUC > 0.8) and correlate with blood-brain barrier disruption, a critical feature of severe SFTS. This distinction is highlighted in the revised manuscript, particularly in the Discussion section , where we emphasize the clinical utility of MMP8 as a peripheral, easily measurable tool for early risk stratification. Furthermore, the robust cross-cohort validation (n = 151) adds significant persuasiveness to our work.

### **Comment 3:** Setting aside the discussion of the mechanisms by which the two single genes are related to severe illness and the occurrence of neurological symptoms, merely considering them as biological markers requires larger samples or even multicenter studies. If FOXJ1 and RGS4 are to have significant implications for the occurrence of neurological symptoms or the progression to severe illness, more basic experiments are needed to confirm this.

**Response:** We appreciate this important feedback. In the revised manuscript, we have reanalyzed the original transcriptomic sequencing data by regrouping the samples according to the presence or absence of neurological symptoms. Based on this reanalysis, we have shifted our focus from FOXJ1 and RGS4 to MMP8, which emerged as a more promising candidate. MMP8 is a well-characterized matrix metalloproteinase with established roles in blood-brain barrier (BBB) disruption and neuroinflammation (as cited in the revised manuscript, references 24-26). Its significant upregulation in fatal cases and strong association with neurological manifestations, such as consciousness disorders and convulsions, suggest a plausible biological mechanism. Although we acknowledge the need for further mechanistic studies, this work establishes MMP8 as a clinically actionable biomarker. To address the limitations of our current mechanistic interpretation and to outline future research directions, including planned studies using SFTS animal models to explore MMP8's role in neuroinvasion, we have added a new section in the Discussion.

### Comment 4: There are already many stable and powerful clinical indicators or combined indicators for predicting neurological symptoms or fatal outcomes. The two new indicators identified in this study lack practical application value.

Response: We appreciate the reviewer for this valuable observation, which is crucial for improving the manuscript. We fully acknowledge that established clinical indicators (e.g., AST, LDH, Cr) and composite scoring systems are widely used for predicting adverse outcomes in SFTS. However, these traditional biomarkers primarily reflect systemic inflammation or organ damage and lack specificity for central nervous system (CNS)-related complications.

Nevertheless, MMP8, which we have identified and validated in two independent cohorts (Zhejiang and Nanjing centers), demonstrates significant correlations with both neurological symptom presence (Fig 4C) and morality(Fig 4D). Specifically, MMP8 achieved area under the curve (**AUC) values of 0.821(95% CI: 0.681–0.962; P=0.006**) and 0.827(95%CI: 0.721-0.932, p<0.001) in the two cohorts, respectively. These AUC values indicate that MMP8 outperforms traditional biomarkers in discriminating patients at high risk of mortality.

Importantly, compared to the reviewer-mentioned traditional biomarkers, MMP8 shows superior discrimination for CNS-related complications due to its mechanistic link to neuroinflammation and blood-brain barrier dysfunction. MMP8 demonstrates both high sensitivity (90.90%) and specificity (58.76%) in predicting mortality, with minimal attenuation in external validation cohorts (Discussion section). These findings establish MMP8 as a novel and specific biomarker for neurological prognosis, addressing a critical gap in current prognostic tools.

**Reviewer #2:**
The manuscript is well-written, and the experimental approach is appropriately designed and executed. Most results are clearly presented and effectively illustrated. However, a major limitation of this study is the lack of mechanistic insight into how upregulated FOXJ1 and downregulated RGS4 in PBMCs contribute to CNS dysfunction and neuroinflammation. Additionally, the study does not provide direct evidence of neuroinflammation. Certain aspects of the paper require further clarification, as outlined below.

**Comment 1:** One of the key concerns of this study is that the two selected biomarkers, FOXJ1 and RGS4, were identified in PBMCs, whereas these genes are primarily expressed and function in the neuronal system. The authors should address this gap by discussing how their findings in PBMCs relate to CNS pathology and whether peripheral gene expression changes can reflect central nervous system processes.

**Response:**We sincerely thank the reviewer for raising this important point regarding the cellular origin and biological relevance of the initially identified biomarkers (FOXJ1 and RGS4). We fully acknowledge that these genes are predominantly expressed in the central nervous system (CNS) and play critical roles in neuronal development and function. Their detection in peripheral blood mononuclear cells (PBMCs) indeed raises questions about their direct relevance to CNS pathology.

In light of this concern and following a careful re-evaluation of our transcriptomic data in conjunction with clinical observations, we have refocused our analysis on MMP8, which emerged as a more promising and mechanistically plausible candidate during our revised investigation. Unlike FOXJ1 and RGS4, MMP8 is highly expressed in peripheral immune cells, particularly neutrophils and macrophages, and has well-established roles in blood-brain barrier (BBB) disruption, neuroinflammation, and secondary brain injury (references 24-26 in the revised manuscript).

We believe that MMP8 serves as a bridge between peripheral immune activation and CNS involvement in SFTS patients with neurological symptoms. Specifically, its upregulation in PBMCs and elevated levels in serum correlate strongly with the presence of neurological manifestations (e.g., consciousness disorders, convulsions)(Fig 4C), suggesting a potential mechanism whereby systemic inflammation contributes to BBB dysfunction and subsequent CNS invasion or damage.

Moreover, we have added a new section in the Discussion to explicitly address the limitations of interpreting peripheral gene expression in the context of CNS pathology. We also outline our future plans to investigate MMP8 dynamics in animal models of SFTS to better understand its role in neuroinvasion and neuropathogenesis.

We appreciate the reviewer's insightful comment, which prompted us to refine our focus toward a more clinically and mechanistically relevant biomarker. We hope this revision adequately addresses the concern.

**Comment 2:**The authors mention clinical stages S1–4, but these need to be briefly defined for the reader. Additionally, patient classification is presented as two groups (S1 vs. S2–4; lines 201–203). Why were S2, S3, and S4 grouped together instead of being analyzed separately? A more precise stage-wise classification could strengthen the conclusions.

**Response:**We appreciate the reviewer's feedback on the clarity and precision of our clinical staging definitions and patient classification strategy.

Severe Fever with Thrombocytopenia Syndrome (SFTS) typically progresses through four distinct stages based on clinical manifestations: S1 (0-7 days, Fever stage), S2 (8-14 days, Deterioration/Organ failure), S3 (15-21 days, Improving), and S4 (22 days and beyond, Convalescence)(References 9). In the revised manuscript, we have added a brief explanation of the clinical stages in the Introduction section (page 5)

Our primary focus was on identifying early prognostic markers during the fever stage (0-7 days, S1). Therefore, we initially distinguished S1 from later stages (S2-S4) , aiming to highlight the critical importance of early neurological symptoms.

**Comment 3:** Gene names FOXJ1 and RGS4 should be italicized throughout the manuscript. The study describes transcriptional changes in these genes but does not validate protein-level expression. The authors should clarify this limitation and, if possible, measure protein levels in PBMCs (or plasma) and cerebrospinal fluid (CSF) to support their findings.

**Response:** We sincerely thank the reviewer for this important suggestion. In the revised manuscript, all gene names are shown in italics. Specifically, MMP8 is consistently italicized throughout the text. Regarding protein-level validation,we have focused on serum MMP8 levels as a clinically accessible and robust proxy. We measured serum concentrations of MMP8 in 151 hospitalized SFTS patients using ELISA. The results demonstrated strong discriminatory performance for predicting mortality (AUC = 0.821 in the first cohort and AUC = 0.827 in the second cohort), with significant correlations to neurological symptoms (e.g., consciousness disorders, convulsions)(Fig 4C).

As cerebrospinal fluid (CSF) samples were not obtained in this study, we were unable to quantify MMP8 alterations in CSF—a limitation explicitly acknowledged in our discussion.

**Comment 4:** Lines 150–152: What was the specific RT-PCR target? If the authors quantified SFTSV RNA, viral RNA load data for all patients should be presented. Were there differences in viral RNA loads between survivors and deceased patients?

**Response:** The SFTSV RNA quantification was performed using real-time RT-PCR targeting the SFTS virus, as previously described (Reference 22 in the revised manuscript). This target is widely used for SFTSV detection due to its high specificity and consistency. Viral RNA quantification was conducted only in the Zhejiang University School of Medicine First Affiliated Hospital cohort (Validation Cohort 2A) due to sample availability and storage conditions. Viral load measurements were not analyzed in Validation Cohort 2B (Nanjing Drum Tower cohort) owing to a missing data rate exceeding 50%. In Cohort 2A, we observed a statistically significant difference in SFTSV RNA loads between survivors and deceased patients (p < 0.001, Mann-Whitney U test). Deceased patients exhibited higher median viral RNA levels (5335632.00/mL vs. 220.68/mL in survivors). These findings are now explicitly stated in the Results section (page 19) and summarized in Table 3.

**Comment 5:** A key finding of this study is that elevated FOXJ1 and decreased RGS4 expression in PBMCs are associated with severe neurological symptoms. However, it remains unclear whether viral replication directly influences these gene expression changes or whether infection-mediated inflammation drives these alterations. The authors should provide viral genome copy numbers for tested samples and discuss potential relationships among viral load, disease severity, transcriptional changes in the two biomarkers, and neuroinflammation.

**Response:** We sincerely thank the reviewer for this important feedback. In the revised manuscript, we have shifted our focus to MMP8 as a more mechanistically and clinically relevant biomarker. Patients with severe neurological symptoms exhibited significantly higher MMP8 mRNA levels compared to those without (p = 0.007, Fig 4C). Furthermore, we discussed that MMP8 expression is potently upregulated by proinflammatory cytokines (e.g., TNF-α, IL-6) characteristic of viral CNS infections. (Line 417-419)

**Comment 6:** In line 206, the authors state that deceased patients were significantly older than recovered patients by approximately 10 years. Given that age is a critical factor in disease severity, the authors should provide a discussion on how age-related changes might influence neurological symptoms and disease progression.

**Response:** We sincerely thank the reviewer for this important observation. In the revised manuscript, we have expanded our analysis to address the potential impact of age on neurological symptoms and disease progression in SFTS patients. We have added a dedicated discussion in the Discussion section (page 27) to explore how age-related changes might contribute to neurological symptoms and disease severity in SFTS patients: Aging is associated with dysregulated immune responses, including reduced T-cell function and elevated pro-inflammatory cytokines (e.g., IL-6, TNF-α). These age-related immune changes may exacerbate systemic inflammation and blood-brain barrier (BBB) disruption, facilitating CNS involvement. On the other hand, older individuals often have pre-existing neurovascular fragility and reduced neuroplasticity, making them more susceptible to viral-induced neuroinflammation and neurological deficits.

Minor Comments

Resolution of Fig 2 and 3 is too low.
In Figure 3C, D, F, and G, labeling the "no-neurological symptom" group as "no-NS" instead of "NNS" would improve clarity.
To enhance readability, tables should visually highlight statistically significant results (e.g., using bold text, underlining, or asterisks). This would help readers quickly identify key findings.

Response: We express sincere gratitude to the reviewer for his/her constructive feedback. All formatting concerns raised have been addressed in the revised manuscript as suggested.

Reviewer #3
**comments 1**:Some figures are not explicitly mentioned in the section where the results are presented, such as some pannels of Figures 2 and 3.

Response: We are grateful for the precise feedback regarding figure citations. All panels—particularly those in Figures 2 and 3—are now systematically referenced in the revised Results section.

**comments 2:** There are references not cited in the text.

Response: We appreciate the reviewer's meticulous attention to citation integrity. All references are now systematically cross-checked and corresponding in-text citations have been ensured throughout the revised manuscript.

**comments 3:** The group of 15 patients is too small to draw generalizable conclusions.

Response: We acknowledge the reviewers' concern regarding the limited sample size in the initial study. In the revised manuscript, we have significantly expanded our validation efforts by collecting serum samples from 151 hospitalized SFTS patients across two independent centers (43 from Zhejiang University First Affiliated Hospital and 108 from Nanjing Drum Tower Hospital). This larger cohort was used to validate the prognostic value of MMP8, a key gene identified in our RNA-seq analysis. The serum MMP8 levels were measured via ELISA, and the results demonstrated robust discriminatory performance for predicting mortality (AUC = 0.821, 95% CI: 0.681-0.962 in the first cohort and AUC = 0.827, 95% CI: 0.721-0.932 in the second cohort). These findings were further supported by multivariate Cox regression analysis, which confirmed MMP8 as an independent predictor of mortality (HR: 1.527, 95% CI: 1.300–1.810). Additionally, we have added detailed pathway enrichment analyses to contextualize the role of MMP8 within broader biological processes (see revised Figures 2 and 4, and Supplementary Material 3).

**comments 4:** Regarding the selection criteria: It is mentioned that the patients were diagnosed with SFTS, but it is not well detailed how the clinical and virological diagnosis was confirmed. Was RT-PCR used in all cases? Were additional clinical criteria included? It is not clear if the 15 patients were randomly selected or if there was any selection bias.

### **Response:** We sincerely thank the reviewer for this important question regarding patient selection and diagnostic criteria.

### **We have added diagnostic criteria for SFTS in **Patients and Samples** section of the revised manuscript. According to China's 2023 Guidelines for Diagnosis and Management of Severe Fever with Thrombocytopenia Syndrome (SFTS), suspected cases are defined by exposure history alongside characteristic clinical presentations (e.g., fever, fatigue, nausea/vomiting). Confirmation requires meeting suspected case criteria plus ≥1 laboratory finding: (1) SFTSV nucleic acid positivity; (2) Viral isolation from plasma; (3) SFTSV-IgG seroconversion or ≥4-fold titer rise in convalescent versus acute-phase sera.**

**comments 5:** Logistic regression is mentioned, but it is not clear whether adjustment was made for variables such as age or comorbidities. Was multicollinearity between variables assessed?

**Response:** Thank you for this insightful comment. In the revised manuscript, we have updated our statistical approach and now primarily employ Cox proportional hazards regression models to assess the association between MMP8 expression levels and patient outcomes, rather than logistic regression. This change allows us to better account for time-to-event data (e.g., survival duration) and provides more clinically relevant insights into prognostic factors. In multivariable Cox regression analyses, we have adjusted for key clinical variables including age, comorbidities (hypertension, diabetes, cardiovascular disease), and baseline laboratory parameters (such as platelet count, D-dimer, AST, ALT, and LDH). These adjustments were made to minimize potential confounding effects.

**comments 6:** In page, 12-13, section "Baseline Characteristics of SFTS Patients", an increase in D-dimer, AST, ALT and LDH in deceased patients is mentioned. Has this been compared with other previous studies? It would be useful to include references.

**Response:** Thank you for this helpful suggestion. The revised manuscript now contextualizes these biomarker elevations through comparison with existing literature in the Discussion (Page 27), with relevant citations added.

**comments 7:** FOXJ1 and RGS4 are mentioned as possible biomarkers. However: FOXJ1: Its role in cilia formation and CSF (cerebrospinal fluid) regulation is interesting, but how does it directly relate to SFTS? RGS4, it is known to be involved in neurological diseases, but it is not mentioned in previous SFTS studies. Is there previous evidence of its role in viral infections?
It is claimed that these biomarkers may be useful in early detection, but the p-values and expression differences are not very large. It is recommended to be more cautious with this claim or to validate the results in an external cohort.

**Response:** We sincerely thank the reviewer for this insightful suggestion. Following re-analysis of our transcriptomic data, we have replaced FOXJ1 and RGS4 with MMP8 as a more robust biomarker for neurological outcomes in SFTS. This decision is supported by two key findings: (1) MMP8 mRNA is significantly upregulated in PBMCs of patients with neurological symptoms compared to those without (adjusted p-value, q<0.05), and (2) serum MMP8 levels were consistently elevated in neurological cases across two independent cohorts (Zhejiang and Nanjing centers; p = 0.0004)(Fig 4C). These results highlight MMP8's potential as a reliable prognostic indicator for neurological complications, with biological relevance to blood-brain barrier disruption and neuroinflammation. Additionally, evidence from prior studies (reference 25) indicates that increased serum MMP8 levels constitute an established predictor of blood-brain barrier injury. We have revised the manuscript to emphasize this finding and its clinical implications.

**comments 8:** In general, I would like to highlight that I find as limitations of this study, like small sample size, the findings need validation in larger studies. Lack of a control group. No comparison with patients with other viral infections or mild SFTS and selection bias, if hospitalized patients were more severe, this could bias the results.

**Respones:** We sincerely thank the reviewer for these valuable comments. Indeeed, the relatively small sample size (n = 15 in the transcriptomic analysis) is a key limitation. In the revised manuscript, we have reanalyzed the original transcriptomic sequencing data by regrouping the samples according to the presence or absence of neurological symptoms. Based on this reanalysis, we have shifted our focus from FOXJ1 and RGS4 to MMP8, which showed significant upregulation in neurological cases (p = 0.0004) and was further validated in two independent cohorts (Zhejiang and Nanjing Hospital). These steps enhance the robustness of our results, but we agree that larger, multi-center studies are essential to confirm the clinical utility of MMP8. As suggested, we have added a dedicated paragraph in the Discussion section (page 28–29) to address the these limitations.
