# Supplementary material for: Neurological manifestations and MMP8 as a prognostic biomarker in severe fever with thrombocytopenia syndrome
Source: PLoS Negl Trop Dis. 2025 Dec 26;19(12):e0013875. doi: 10.1371/journal.pntd.0013875 (PMC12758824; doi:10.1371/journal.pntd.0013875)
Supplement: Attachment — Submitted filename: Response_to_Reviewers_auresp_1.docx [file pntd.0013875.s010.docx]

**Response to Reviewers’ Comments**

**Manuscript number:** PNTD-D-25-01139

**Title:** Neurological manifestations and MMP8 as a prognostic biomarker in Severe Fever with Thrombocytopenia Syndrome

**Dear Editors and Reviewers,**

We sincerely appreciate the opportunity to revise our manuscript entitled “**Neurological manifestations and MMP8 as a prognostic biomarker in Severe Fever with Thrombocytopenia Syndrome**”. We are grateful for your thoughtful and constructive comments, which have greatly enhanced the clarity, rigor, and overall quality of our work and provided valuable guidance for our research.

We have carefully considered each point raised and have provided detailed, point-by-point responses below. To facilitate your review, our replies are presented in blue font. Text quoted from our manuscript is shown in *italics*, with newly added or revised text highlighted in *blue italics*. Following each response, we have included the corresponding revised section from the manuscript for your convenience. All changes have also been incorporated into the main revised manuscript and are highlighted in red font. We believe the manuscript is now substantially improved.

Thank you once again for your time and consideration.

**Point-by-Point Responses to Reviewers' Comments**

**Reviewer #1:**

This study aims to explore relevant mechanisms and identify biomarkers of SFTS. This study presents major shortcomings in clinical relevance, research logic, and experimental design. Firstly, the central research question fails to address a real clinical need, and the identified biomarker lacks adequate comparative analysis and validation. Secondly, the study design suffers from issues such as inappropriate sample selection, unclear sampling time points, and a disconnect between experimental strategies and research objectives. These factors collectively undermine the reliability of the findings and limit their translational value in clinical practice.

**Comment 1:** Over the past several years, numerous studies have explored biomarkers associated with severe SFTS. In fact, many routine hematological parameters—such as APTT and D-dimer, as mentioned in the current manuscript—are already useful in assessing disease progression. Clinicians can often extract meaningful diagnostic clues from standard biochemical monitoring. This raises the question: is there a genuine need to search for additional biomarkers? Unless such biomarkers can effectively distinguish SFTS from other thrombocytopenia-related diseases or enable early identification in the absence of accessible nucleic acid testing, their clinical value may be limited. Therefore, I believe this study does not sufficiently address a practical clinical problem.

**Response:** We sincerely thank you for this thoughtful question, which gets to the heart of our study's clinical motivation. To demonstrate the practical clinical value of our findings, we would like to highlight three key contributions of our study.

1. **A Non-Invasive Bedside Tool for Early Warning**: First, our study provides a direct, non-invasive tool for immediate risk stratification. We found that specific, easily observable neurological symptoms (such as consciousness disorder) are strongly associated with poor prognosis. This allows clinicians to make a rapid initial risk assessment at the bedside, without waiting for a blood draw, and to heighten their vigilance for high-risk patients from the moment of admission.
2. **MMP8 Offers Superior Prognostic Accuracy**: Second, to directly address your point about the utility of existing markers, we have now updated **Figure 6** to include a direct comparison of prognostic performance. As the new figure shows, the accuracy of MMP8 as a single biomarker for predicting mortality (AUC=0.827) is substantially higher than that of both D-dimer (AUC=0.701) and APTT (AUC=0.726). This provides clear, data-driven evidence that MMP8 offers superior prognostic information beyond these routine tests.
3. **An Integrated Model with the Highest Predictive Power**: Finally, we show that the greatest clinical utility is achieved by integrating MMP8 with conventional markers. Our novel **MCCD** model achieves the highest predictive accuracy of all (AUC=0.843), outperforming any single biomarker. This demonstrates that MMP8 is not just another marker but a synergistic component that creates a much more powerful and clinically actionable tool for predicting mortality.

In summary, our study provides a multi-faceted approach—combining clinical signs, a superior standalone biomarker, and an optimized predictive model—to address the specific clinical problem of identifying high-risk SFTS patients prone to fatal outcomes. To better articulate these contributions, we have now incorporated this three-point summary into the conclusion of our **Discussion** section (Page 36-37, Lines 606-611). We are very grateful for your insightful comment, which has pushed us to better articulate the significance of our findings with new, direct evidence.

(Page 36-37, Lines 606-611):

*This multi-faceted approach offers significant clinical advantages: clinicians can use neurological symptoms for immediate, non-invasive risk stratification at the bedside. Furthermore, MMP8 as a standalone biomarker demonstrates superior prognostic performance over traditional indicators like APTT and D-dimer; and the MMP8-inclusive MCCD model provides the highest overall predictive accuracy.*

**Comment 2:** If the goal is to identify biomarkers predictive of disease progression, then the study design should clearly define the timing of sample collection relative to disease onset. Although the authors mention different disease phases in the Methods section, they fail to specify which phase their samples correspond to or whether sample collection was standardized. Furthermore, PBMCs may not be the most appropriate sample type for this purpose. For translational biomarker discovery, serum samples would be more suitable. Using PBMCs to identify gene expression changes may not yield clinically actionable results. As such, the study's rationale appears disorganized and its objectives unclear.

**Response:** We appreciate the reviewer's feedback concerning the clarity of our study design and methodology. We apologize that our initial description was unclear, and we thank you for the opportunity to better articulate the logical progression of our research. Our study employed a deliberate two-stage design:

1. **Discovery Phase** **(Mechanistic Exploration using PBMCs):**

Since severe SFTS is characterized by a strong systemic inflammatory response and cytokine dysregulation, we chose PBMC RNA-sequencing as an unbiased and powerful tool to explore the underlying transcriptomic mechanisms of this host immune response and generate novel hypotheses.

2. **Validation Phase (Clinical Translation to a Serum Biomarker):**

However, we fully agree with your point that for a biomarker to have translational value, it must be easily accessible in a clinical setting. Therefore, the validation phase of our study was designed specifically to bridge this gap from mechanistic discovery to a practical clinical tool. We validated our lead candidate, MMP8, at the protein level in serum samples—a much more accessible and clinically relevant medium. This validation was performed in two large, independent patient cohorts to ensure the robustness of our findings.

Regarding the crucial point of sample timing, following your valuable suggestion, we have significantly clarified this in the **Methods** section (Page 7, Lines 137-141). The revised text now specifies that an initial sample was collected from all 15 patients in the discovery cohort within 24 hours of their hospital admission, with subsequent longitudinal sampling performed on a subset to capture disease dynamics.

We hope this more detailed explanation clarifies our rationale and demonstrates the organized, two-phase design of our study, moving logically from a mechanistic question using PBMCs to a clinically practical answer in serum. We are grateful for your guidance, which has helped us to significantly improve the manuscript.

(Page 7, Lines 137-141):

*An initial blood sample was collected from all 15 patients within 24 hours of hospital admission. To capture dynamic gene expression changes, longitudinal samples were subsequently collected from a subset of these patients (3 recovered and 1 deceased) at different stages of the disease (S1, S2, and S3).*

**Comment 3:** The identification of MMP8 as a biomarker appears insufficiently substantiated. In biomarker research, it is common to propose several candidate molecules and validate them in an independent cohort. However, this study does not report any additional candidates or describe a comprehensive validation process. Even if such work was performed, a rigorous study should include those results. Additionally, neurological symptoms were not described in cohort 1, yet this cohort was used for screening biomarkers associated with neurological manifestations. This discrepancy raises concerns about the appropriateness of the cohort design and the interpretation of findings.

**Response:** We sincerely thank you for this critical feedback, which has pushed us to better explain the rationale and rigor of our biomarker selection and validation process. We agree completely that a comprehensive validation process is essential, and we are grateful for the opportunity to clarify our methodology.

Regarding the substantiation of MMP8 as a biomarker, our approach to identifying MMP8 was systematic and data-driven.

1. **Initial Discovery**: To find candidates specifically linked to the fatal neurological symptoms and subsequent adverse outcomes, we first performed a transcriptomic analysis comparing SFTS patients with lethal neurological symptoms to those without. This unbiased screen yielded eight significantly upregulated genes, with MMP8 being one of them.
2. **Candidate Prioritization**: Upon evaluating these eight candidates for their potential as clinically viable serum biomarkers, we found that the other seven genes all had very low expression levels (mean FPKM < 5) (Page 22, Lines 376-380). This made them poor candidates for further study, as their protein products would be unlikely to be reliably detectable in serum. This data-driven filtering positioned MMP8 as our single most promising and viable candidate from the initial transcriptomic discovery.
3. **Validation with a Parallel Candidate** **(MMP9)**: To further strengthen our validation process, we have sought a logical parallel candidate. Our selection of this candidate was guided by a literature review, which pointed to MMP9. Like MMP8, MMP9 is a member of the matrix metalloproteinase family and is also involved in blood-brain barrier disruption, making it a highly relevant molecule for comparison in the context of neurological complications.

We had measured serum levels for both markers in our original study. However, we initially omitted the MMP9 data for brevity because the results were not significant.

We now recognize that including these negative findings is crucial for transparency and significantly strengthens our conclusions. Following your valuable suggestion, we have decided to include this important negative data. The analysis showing that, in stark contrast to MMP8, serum MMP9 levels were not significantly different across any patient groups, has now been added to the **Results section** (Page 24, Lines 405-409) and is presented in a new **Supplementary Figure S3**. This addition serves to highlight the specificity of MMP8 as a prognostic marker in SFTS.

Regarding the cohort design and neurological symptoms: We would like to respectfully clarify a potential point of confusion regarding the cohort descriptions. The initial biomarker discovery (RNA-seq) was performed on the **Discovery Cohort** (n=15). The detailed neurological symptoms for this specific cohort were indeed collected and are presented in **Table 2**. **The Validation Cohort 1** (n=103), a larger cohort, was then used to specifically validate the prognostic significance of these neurological symptoms, with the full data presented in **Table 3**. Therefore, the cohort used for screening biomarkers was fully characterized for neurological manifestations. We apologize if our initial description of the multi-cohort design was not sufficiently clear.

We thank you again for your insightful comment, which has helped us to improve the quality and clarity of our manuscript.

(Page 22, Lines 376-380):

*Among the 8 upregulated genes, after filtering out low expression genes (mean FPKM <5), we prioritized MMP8(matrix metallopeptidase 8) for further scrutiny due to its established roles in inflammatory cascades and extracellular matrix remodeling (Fig 4B) (Table S2).*

(Page 24, Lines 405-409):

*To further investigate the specificity of MMP8 as a biomarker, we also measured serum levels of MMP9, another metalloproteinase implicated in blood-brain barrier disruption. In contrast to MMP8, our analysis revealed that MMP9 concentrations were not significantly different between deceased and recovered patients, nor between patients with or without neurological symptoms (Fig S3).*

**Reviewer #2:**

In this manuscript, through prospective and retrospective cohort studies, the authors demonstrated that neurological symptoms during the early stages of SFTS might serve as a predictor of fatal outcomes, and also revealed MMP8 as a potential biomarker for SFTS prognosis.

**Comment 1**: Figure 4C, 4D and Fig S1, the authors detected and compared the MMP8 levels in the serum of SFTS patients. A comparison with healthy controls should also be included.

**Response:** We are very grateful to the reviewer for this important suggestion. We completely agree that including a comparison with healthy controls provides a critical baseline and significantly strengthens our findings.

Following this valuable advice, we have now recruited a cohort of 19 healthy individuals, measured their serum MMP8 levels, and incorporated this data into our analyses. We believe this addition provides a much clearer context for interpreting the elevated MMP8 levels observed in SFTS patients. We have updated Figure 4C, Figure 4D, and Figure S1 to include the healthy control group data, allowing for a direct statistical comparison with our patient groups. The corresponding figure legends (Page 26, Line 445-447) and the main text in the **Results** section have also been revised to reflect these new and important findings (Page 23, Line 388-398).

(Page 23, Line 388-398):

***The results showed that MMP8 concentrations were significantly elevated only in the patient group with lethal neurological symptoms (consciousness disorder or convulsion). Their levels were significantly higher than both the levels in patients without such symptoms (p=0.001) and in the healthy control group (p=0.019). Notably, there was no significant difference in MMP8 levels between SFTS patients without neurological symptoms and the healthy controls. This finding indicates that MMP8 elevation is specifically associated with the onset of severe neurological manifestations. This trend was significantly replicated in the larger Validation Cohort 2B (Fig S2B). Although a similar pattern of MMP8 elevation was observed in Validation Cohort 2A, it did not reach statistical significance, potentially due to the smaller sample size of this subgroup (n=43) and the consequently limited statistical power (Fig S2A).***

**Comment 2:** Please analyze the correlation between viral load and MMP8 levels in the serum.

**Response:** We are very grateful to the reviewer for this insightful suggestion to explore the relationship between viral replication and MMP8 levels. We agree that this is an important analysis for understanding the potential mechanisms of MMP8 upregulation.

Following your recommendation, we performed a Spearman correlation analysis between serum MMP8 levels and viral load in the patients from Validation Cohort 2A, for whom both data points were available. Our analysis did not reveal a statistically significant correlation between these two variables (Spearman's r= 0.233, p= 0.133).

We believe this is an interesting finding in itself. We speculate that it may suggest that serum MMP8 is a marker of the host's downstream immunopathological response (for example, the cytokine storm) rather than a direct correlate of viral replication at a given time point. It is possible that the systemic inflammation triggered by the virus is the more direct driver of MMP8 expression.

We have added a brief description of this analysis and its result in the **Results** section (Page 25-26, Line 430-436). Furthermore, we have expanded our **Discussion** to explore the potential implications of this non-significant finding, proposing that MMP8 likely reflects the severity of the host's inflammatory cascade (Page 32, Line 531-535). We have also added a scatter plot of the analysis as a new supplementary figure (**Figure S4**).

(Page 25-26, Line 430-436):

***Given the strong association between serum MMP8 and adverse clinical outcomes, we next sought to explore potential drivers of its upregulation. To investigate whether MMP8 expression was directly associated with viral replication, we analyzed the correlation between serum MMP8 concentrations and SFTSV viral load. The analysis revealed no statistically significant correlation between the two variables (Spearman's r=0.233, p=0.133) (Fig S4). This suggests that the elevation of MMP8 may be driven by downstream inflammatory pathways rather than directly by the viral burden itself.***

(Page 32, Line 531-535):

***Reinforcing its role as a marker of the host's immunopathology, our analysis found no direct correlation between serum MMP8 levels and SFTSV viral load. This finding suggests that MMP8 upregulation is not driven by the level of viral replication itself but is rather a consequence of the broader, systemic inflammatory cascade that characterizes severe SFTS.***

**Comment 3:** Has MMP8 been considered as biomarker in other infectious diseases? The authors should discuss this.

**Response:** Thank you for prompting us to place our findings in a broader context. This is an important point that enhances the scope of our discussion. We have added a new paragraph to the **Discussion** section (Page 32, Lines 536-541) to contextualize our work within the existing literature on infectious diseases. This new section now discusses key studies on the role of MMP8 in other severe infections. Specifically:

1. We now cite research proposing MMP8 as a potential prognostic biomarker in pediatric septic shock, where its levels directly correlate with organ failure and mortality.
2. We also discuss its well-established mechanistic link to the breakdown of the blood-brain barrier in cerebral malaria.

We believe this addition better frames the novelty and significance of our study. It allows us to show that our work builds upon previous findings in bacterial sepsis and parasitic diseases, while highlighting our unique contribution: validating MMP8 for the first time as a robust prognostic biomarker specifically in a viral hemorrhagic fever (SFTS) and linking it directly to the fatal neurological complications.

(Page 32, Lines 536-541):

***Indeed, the potential of MMP8 as a biomarker for severity in infectious diseases is an emerging area of research. For example, in pediatric septic shock, MMP8 levels were found to directly correlate with organ failure and mortality, leading to its proposal as a potential prognostic biomarker for bacterial sepsis****^[30]^****. In the context of cerebral malaria, while not explicitly validated as a prognostic marker, its upregulation is considered a key mechanistic factor in the breakdown of the blood-brain barrier****^[31]^****.***

**Comment 4:** Figure legends for Figures 3, 4 and Fig S1 must include descriptions of the statistical methods employed.

**Response:** We appreciate the reviewer for spotting this omission. We apologize for the lack of detail in the original figure legends. We have carefully revised the legends for **Figure 3, Figure 4, and Figure S1** to explicitly state the statistical tests used for each comparison (e.g., Fisher's exact test, Kruskal-Wallis test). (Page 21, Line 358-364) (Page 26, Line 447-448) (Page 38, Line 637-648)

**Reviewer #3:**

The objectives of this study are clearly articulated. The authors sought to (i) evaluate the prognostic significance of neurological symptoms in SFTS patients, (ii) explore underlying molecular mechanisms using transcriptomics, and (iii) validate MMP8 as a prognostic biomarker in multiple independent cohorts.

While an explicit hypothesis statement (“We hypothesize that neurological symptoms in SFTS patients are linked to transcriptomic alterations and that MMP8 is an independent prognostic biomarker”) could be more clearly stated, the research question is inherently testable and consistently pursued throughout the manuscript. I have following comments:

**Comment 1:** Diagnosis followed standardized Chinese national guidelines, and inclusion/exclusion criteria were appropriate. The demographic description (age, comorbidities, baseline labs) is well detailed.

The population aligns well with the hypothesis (prognostic biomarkers in SFTS). However, generalizability outside Chinese populations (e.g., Japan, South Korea) remains untested and should be acknowledged.

**Response:** We thank the reviewer for the positive assessment of our study population and methodology. We fully agree with the comment on generalizability. Following your valuable suggestion, we have added a statement to the limitations paragraph in the **Discussion** section (Page 34, Line 569-572), acknowledging that our study was conducted exclusively in Chinese patients and that our findings require validation in other populations where SFTS is endemic, such as those in Japan and South Korea.

(Page 34, Line 569-572):

***First, the study cohort consisted exclusively of Chinese patients, which potentially limits the generalizability of our findings. Validation in other SFTS-endemic populations, such as those in Japan and South Korea, is therefore essential.***

**Comment 2:** RNA-seq cohort (n=15, 4 deaths): Too small to provide robust statistical power for high-dimensional transcriptomic analysis. This limits confidence in differentially expressed gene calls and enrichment results. Authors should highlight this limitation more strongly.

**Response:** We sincerely thank the reviewer for this critical and valid point. We completely agree that the small sample size of our initial transcriptomic cohort (n=15) is a primary limitation and that this needs to be highlighted more strongly and transparently in our manuscript.

To address this concern, we have substantially revised the Limitations section of our **Discussion** (Page 35, Lines 575-581). In the revised text, we now explicitly state:

1. That the small cohort size limits the statistical power, which in turn reduces the confidence in the differentially expressed gene (DEG) calls and the robustness of the enrichment analyses.
2. We have clearly framed this part of our study as an exploratory, hypothesis-generating phase, rather than a conclusive analysis.
3. We emphasize that our study was designed to mitigate this very issue. We used the transcriptomic data primarily as a discovery tool and then successfully validated our key candidate, MMP8, in two independent and much larger clinical cohorts (Validation Cohort 2A, n=43, Validation Cohort 2B, n=108).

(Page 35, Lines 575-581):

***Second, a primary limitation is the small cohort size for the initial PBMC transcriptomic analysis, which constrained its statistical power. Therefore, we appropriately treated this transcriptomic analysis as an exploratory, hypothesis-generating phase of our research. Our confidence in the primary finding is substantially bolstered by their successful validation in larger, independent cohorts (n=103 and n=151), which confirmed the prognostic significance of neurological symptoms and serum MMP8 levels.***

**Comment 3:** Small RNA-seq sample size may produce false positives. Authors should consider FDR-adjusted thresholds in DEGs instead of relying solely on p<0.05

**Response:** We sincerely thank the reviewer for this critical and highly constructive suggestion. We completely agree that using an FDR-adjusted threshold is a more rigorous approach for our transcriptomic analysis, and we have acted on this advice.

In our revised manuscript, we have retained our original analysis for consistency with the existing figures, but we have now added a supplementary analysis using the more stringent q-value<0.05 threshold. We have updated the **Results section** (Page 15, Lines 245-249) to include a summary of these new findings, noting that this stricter analysis identified 1693 DEGs and, reassuringly, confirmed the significant enrichment of the nervous system development pathway.

(Page 15, Lines 245-249):

***Given the small sample size, we also performed a more stringent analysis using an FDR-adjusted p-value (q-value<0.05) (Fig S1), which identified 1693 DEGs (611 upregulated, 1082 downregulated). Reassuringly, GO analysis of this stricter gene set also revealed a significant enrichment of the nervous system development pathway.***

**Comment 4:** Minor text edits: Correct typographical errors (“datas” → “data”)

**Response:** Thank you for your careful proofreading. We apologize for this grammatical error. We have corrected all instances of "datas" to "data" throughout the entire manuscript (e.g. Page 28).

(Page 28):

*Data are median (IQR) or n (%) or mean ± standard deviation. P values were calculated by two-sided Mann-Whitney U test, t test or Pearson's chi-squared test according to the normal distribution of ~~datas~~ data.*

**Comment 5:** Since neurological symptoms are central to the study, absence of cerebrospinal fluid MMP8 (or other neuroinflammation markers) is a missed opportunity, if samples still available, it would be helpful to add this data too.

**Response:** We agree wholeheartedly that CSF analysis would have provided direct evidence of neuroinflammation. Unfortunately, CSF samples were not available for MMP8 analysis in this study due to the invasive nature of lumbar puncture and associated clinical constraints. We have acknowledged this as a limitation in the **Discussion** section. We have also cited literature suggesting that in some CNS infections, serum *MMP8* can be as good or even better than CSF *MMP8* at predicting blood-brain barrier disruption (Page 35, Line 589-596), which provides some support for our serum-based approach.

(Page 35, Line 589-596):

***Additionally, ~~Another limitation is~~ the absence of cerebrospinal fluid (CSF) samples~~, precluding~~ precluded the direct measurement of CSF MMP8 levels. However, prior evidence suggests that serum MMP8 exhibits stronger predictive value for blood-brain barrier (BBB) disruption than CSF MMP8 in tick-borne encephalitis (TBE) patients^[28]^. Therefore, future prospective studies with both serial serum and CSF sampling are warranted to validate these dynamic patterns and directly investigate the link between systemic and CNS MMP8 expression.***

**Comment 6:** Author should also add the recent reports on the presence of this virus from Kenya and Pakistan.

**Response:** We sincerely thank the reviewer for this important suggestion. We agree with the reviewer that the geographic scope of SFTSV and related viruses is expanding beyond East Asia, and our introduction did not reflect these recent findings. We have now updated the manuscript to include the latest reports on serological evidence of SFTSV or related viruses in Pakistan and, more recently, in Kenya, underscoring the growing global relevance of this pathogen. The appropriate citations have been added to the revised sentence in the **Introduction** section (Page 5, Line 94-96).

(Page 5, Line 94-96):

***Furthermore, recent serological evidence suggests its geographic scope is expanding, with related viruses also being reported in Pakistan****^[9]^* ***and Kenya****^[10]^****.***

**Comment 7:** Dabie bandavirus (DBV) is the new name of SFTSV, author should mention like this while mentioning the old name.

**Response:** We sincerely thank the reviewer for this important and timely suggestion regarding the updated virus nomenclature. We have revised the manuscript accordingly. At the first mention of the virus in the **Introduction section** (Page 4, Line 86-90), we now introduce it by its official name, Dabie bandavirus (DBV), and clarify that it is also known as the SFTS virus (SFTSV). We appreciate the reviewer's help in ensuring our manuscript uses the most current and accurate terminology.

(Page 4, Line 86-90):

***Severe Fever with Thrombocytopenia Syndrome (SFTS) is an emerging tick-borne infectious disease caused by the Dabie bandavirus (DBV), also known as the SFTS virus (SFTSV). It is classified in the Banyangvirus genus, within the Phenuiviridae family and the Bunyavirales order~~, a member of the Phlebovirus genus in the Bunyaviridae family~~****^[1,2]^****.***

**Comment 8:** Also Bunyaviridae family has been updated to Bunyavirale order, so author should check the recent ICTV classification to update in the manuscript.

**Response:** We are grateful to the reviewer for pointing out the need to update the virus's taxonomic classification based on the latest ICTV guidelines. We agree this is a critical detail. We have now corrected the taxonomy in the **Introduction section** (Page 4, Line 86-90). The text now reflects that the Bunyaviridae family has been elevated to the order Bunyavirales, and we have updated the virus's full classification within this new order (Banyangvirus genus, Phenuiviridae family). Thank you for helping us ensure the accuracy of this information.

(Page 4, Line 86-90):

***Severe Fever with Thrombocytopenia Syndrome (SFTS) is an emerging tick-borne infectious disease caused by the Dabie bandavirus (DBV), also known as the SFTS virus (SFTSV). It is classified in the Banyangvirus genus, within the Phenuiviridae family and the Bunyavirales order~~, a member of the Phlebovirus genus in the Bunyaviridae family~~****^[1,2]^****.***

**Reviewer #4:**

The manuscript presents a well-designed and comprehensive study with substantial clinical significance. By employing a multicenter, multi-cohort design, the authors systematically examined the relationship between neurological symptoms and adverse outcomes in patients with SFTS. Importantly, the study successfully identified and validated matrix metalloproteinase-8 (MMP8) as a potential prognostic biomarker associated with neurological injury. The research follows a clear and coherent logic, progressing from clinical observations (neurological symptoms), to mechanistic exploration using transcriptomics, back to large-scale clinical validation with serum samples, and finally culminating in the development and validation of a multi-parameter prognostic model (MCCD).

Overall, the manuscript is of high scientific value, supported by robust data, and presents credible conclusions. Nevertheless, certain aspects would benefit from further clarification and refinement, as detailed below.

**Comment 1:** The manuscript mentions neurological symptoms such as “consciousness disorder,” “restlessness,” and “mental state change.” It is recommended to provide the specific assessment criteria for these symptoms in the Methods section. For example, was “consciousness disorder” quantified using standardized tools such as the Glasgow Coma Scale (GCS)? This is crucial for ensuring the objectivity and reproducibility of the results.

**Response:** We are very grateful to the reviewer for this crucial suggestion. We completely agree that providing specific, objective assessment criteria for the neurological symptoms is essential for the study's reproducibility and scientific rigor.

We have now added a new subsection in the **Methods section** titled "Clinical Assessment of Neurological Symptoms" (Page 9, Lines 169-175) to explicitly define these terms. The specific criteria we have added are:

1. **Consciousness Disorder**: We have defined this as a moderate-to-severe impairment of consciousness, corresponding to a Glasgow Coma Scale (GCS) score of 12 or less.
2. **Restlessness**: We have defined this using the Richmond Agitation-Sedation Scale (RASS), where restlessness corresponds to a score of +1.
3. **Mental State Change**: We have clarified this term as apathy, characterized by a diminished motivation and reduced spontaneous interaction with the surroundings.

(Page 9, Lines 169-175):

***Clinical Assessment of Neurological Symptoms Neurological symptoms were systematically recorded throughout the clinical course. Consciousness Disorder: This was defined as a moderate-to-severe impairment of consciousness, corresponding to a Glasgow Coma Scale (GCS) score of 12 or less. Restlessness: This was defined as agitation corresponding to a Richmond Agitation-Sedation Scale (RASS) score of +1. Mental State Change: This was primarily defined as apathy, characterized by a diminished motivation and reduced spontaneous interaction with the surroundings.***

**Comment 2:** Sample collection time points: For the RNA-seq samples, it was stated that they were collected during hospitalization. Could more specific information be provided—for instance, were all samples collected within 24 hours of admission, or were they collected at different stages of the disease (e.g., S1, S2)? This detail is important for interpreting the dynamic changes in gene expression.

**Response:** Thank you for this excellent point. We agree that providing precise details on the sample collection timing is critical for the interpretation of our transcriptomic data. To address this, we have revised the **Participants and samples** subsection within the **Methods** section (Page 7, Lines 137-141) to provide a clearer description. We have now specified that:

1. An initial blood sample for RNA-seq was collected from all 15 patients within 24 hours of their admission to the hospital.
2. To analyze the dynamic nature of gene expression, we performed longitudinal sampling on a subset of patients (three who recovered and one who died) at various stages of the disease (S1, S2, and S3).

(Page 7, Lines 137-141):

***An initial blood sample was collected from all 15 patients within 24 hours of hospital admission. To capture dynamic gene expression changes, longitudinal samples were subsequently collected from a subset of these patients (3 recovered and 1 deceased) at different stages of the disease (S1, S2, and S3).***

**Comment 3:** In the Cox regression analysis, how were the candidate variables selected for inclusion in the multivariable model? Were they based on variables with p < 0.05 in the univariable analysis, or determined by clinical expertise? It is suggested to clarify this point.

**Response:** We thank the reviewer for this important question and for prompting us to improve the clarity of our methodology. In response to your suggestion, we have revised the **Statistical analysis** subsection of the **Methods** section (Page 11, Lines 210-212) to explicitly state the variable selection criteria. We believe this addition makes our methods section more transparent, and we are grateful for the feedback.

(Page 11, Lines 210-212):

***Multivariate analysis was performed using Cox proportional hazards regression models, with candidate variables selected via a forward stepwise method (entry P<0.10).***

**Results**

**Comment 4:** Figure 2 presents a large amount of transcriptomic data. It is recommended that the figure legend provide more detailed descriptions of the purpose and main findings of each subpanel (A–I) to help readers better understand the results.

**Response:** We thank the reviewer for this constructive feedback. We completely agree that a more detailed figure legend is essential for guiding readers through the complex transcriptomic data presented in **Figure 2**.

To address this, we have substantially revised the legend for **Figure 2**. The new, expanded legend now provides a clear narrative for each subpanel (A through I) by describing its specific purpose, summarizing its main finding, and clarifying key visual elements. (Page 16-17, Line 270-307)

**Comment 5:** In the description of the MCCD model, the formula MCCD = 0.058 × MMP8 + 0.001 × CK + 0.041 × Cr + 0.229 × D-dimer is provided. What are the units of each variable? For example, is MMP8 expressed in ng/mL, CK in U/L, etc.? It is suggested to specify the units either alongside the formula or in the main text to facilitate reader interpretation and application.

**Response:** We thank the reviewer for this very practical and important suggestion. We agree that specifying the units for each variable in the MCCD model is essential for the formula's interpretation and potential application by other researchers.

To address this, we have revised the **Results** section (Page 28, Lines 462-465) to include the units directly within the prognostic formula.

(Page 28, Lines 462-465):

***The final prognostic index was expressed as MCCD = 0.058 × MMP8 (ng/mL) + 0.001 × CK (U/L) + 0.041 × Cr (μmol/L) + 0.229 × D-dimer (mg/L FEU), representing a linear predictor of log-hazard ratios.***

**Conclusions:**
**Comment 6:** The discussion section could further elaborate on the specific clinical implications of the study findings. For instance, when clinicians detect elevated MMP8 levels in SFTS patients (e.g., exceeding 18.69 ng/mL) accompanied by early signs of altered consciousness, what specific “intensified therapeutic interventions” (line 440) should be implemented? For example, should immediate transfer to the ICU be recommended, should closer monitoring of vital signs be performed, or should certain experimental therapies targeting inflammation or blood–brain barrier protection be considered? Providing more concrete recommendations would greatly enhance the translational value of the manuscript.

**Response:** We thank the reviewer for this insightful suggestion to enhance the translational value of our findings. We agree that providing more concrete clinical recommendations is important.

Following your valuable suggestion, we have revised the **Discussion** section (Page 31, Lines 516-521) to elaborate on what "intensified therapeutic interventions" would entail. We now specify that for high-risk patients (e.g., those with elevated MMP8 and early neurological symptoms), these interventions include early consideration for ICU admission for close monitoring and proactive management of organ dysfunction. We have also added that our findings provide a strong rationale for future clinical trials to investigate therapies targeting MMP8 or blood-brain barrier protection.

(Page 31, Lines 516-521):

***Specifically, such interventions include early consideration for admission to an intensive care unit (ICU) for continuous monitoring and proactive management of key complications like coagulopathy and organ dysfunction. Furthermore, our findings suggest that therapies targeting MMP8 or protecting the blood-brain barrier warrant investigation in future clinical trials for these high-risk patients.***

**Comment 7:** The authors mention that future prospective studies are needed to determine whether the peak of MMP8 occurs during the S1 stage. Based on the existing data, some preliminary inferences could be made. For example, among the 15 patients in the discovery cohort, are there multi-time-point samples that could provide an initial depiction of the dynamic trend of serum MMP8 changes? Even if the data are limited, such findings could serve as preliminary evidence and be included in the discussion.

**Response:** We thank the reviewer for this excellent suggestion to explore the dynamic trends within our existing data. We agree that even a preliminary analysis could provide valuable insights.

Following this advice, we performed a longitudinal analysis of serum MMP8 levels for the four patients in our discovery cohort who had samples collected at multiple time points (S1, S2, and S3). The results, along with a detailed descriptive legend (Page 39, Lines 659-665), are now presented in a new **Supplementary Figure S5**, and a descriptive analysis has been added to the **Discussion** **section** (Page 35, Lines 585-589).

Our descriptive analysis highlights a clear distinction: the single patient with a fatal outcome showed a markedly and progressively rising trajectory to a very high peak MMP8 level. In contrast, the three patients who recovered displayed more heterogeneous dynamic patterns.

While we explicitly state that the small sample size precludes formal statistical testing, we believe this descriptive analysis provides valuable preliminary evidence, as suggested by the reviewer. It strengthens the rationale for future prospective studies to investigate the trajectory of MMP8 as a prognostic indicator. We are grateful for the guidance to include this analysis.

(Page 35, Lines 585-589):

***Another limitation is the lack of dynamic MMP8 analysis in our main cohorts. To preliminarily explore this, we analyzed longitudinal samples from four patients (3 recovered and 1 deceased) (Fig S5). Despite the small sample size, a clear distinction emerged: the single fatal case showed a progressively rising trajectory, whereas the three recovered patients showed variable trends.***
