# Supplementary material for: Neurological manifestations and MMP8 as a prognostic biomarker in severe fever with thrombocytopenia syndrome
Source: PLoS Negl Trop Dis. 2025 Dec 26;19(12):e0013875. doi: 10.1371/journal.pntd.0013875 (PMC12758824; doi:10.1371/journal.pntd.0013875)
Supplement: Attachment — Submitted filename: Response_to_Reviewers_auresp_2.docx [file pntd.0013875.s011.docx]

**Response to Reviewers’ Comments**

**Manuscript number:** PNTD-D-25-01139

**Title:** Neurological manifestations and MMP8 as a prognostic biomarker in Severe Fever with Thrombocytopenia Syndrome

**Dear Editors and Reviewers,**

We sincerely appreciate the opportunity to submit a revised version of our manuscript and thank you for your constructive comments and helpful suggestions. We have carefully revised the manuscript accordingly. Below we provide a point-by-point response to the journal requirements and reviewer comments. Changes have also been incorporated into the main revised manuscript and are highlighted in red font.

All changes mentioned below have been incorporated into both the clean and tracked-changes versions of the revised manuscript.

**Journal Requirements**

**1) Clarification of the Ethics Statement and type of consent**

**Comment:** Please state whether the consent obtained is verbal or written.

**Response:** We thank the Editors for this important reminder. In this study, written informed consent was obtained from all patients or their immediate relatives prior to sample and data collection and for their use in research. We have revised the Ethics Statement in the manuscript to explicitly state this.

(Page 8, Lines 156-160):

***Ethics Statement This study was approved by the Clinical Research Ethics Committee of the First Affiliated Hospital, College of Medicine, Zhejiang University (approval number 2022–119) and complied with the principles of the Declaration of Helsinki.*** *Written i****~~I~~nformed consent was obtained from all patients or their immediate relatives for sample and data collection, as well as their use in research.***

**2) Data availability for accession HRA010122**

**Comment:** The data under accession HRA010122 were indicated as being available in the future. Please ensure that the dataset will be made freely accessible in accordance with the journal’s open data policy, or explain why an exemption is needed.

**Response:** We fully agree with the journal’s open data policy. We have now released the dataset under accession HRA010122 without embargo, so that it is freely accessible to readers and reviewers.

**Point-by-Point Responses to Reviewers' Comments**

**Reviewer #2:**

**Comment 1:** The quality of the figures needs to be optimized, and the annotations should be standardized, especially in Fig. 4, Fig. 6 and Fig. S2.

**Response:** We appreciate this helpful suggestion. In the revised submission, we have re-exported and optimized all figures, with particular attention to Fig. 4, Fig. 6 and Fig. S2. We improved resolution, adjusted font sizes, and standardized labels, axis titles, abbreviations, and panel annotations to ensure consistency throughout the manuscript. We believe that the visual quality and readability of all figures have been clearly enhanced.

**Comment 2:** In Fig. S2, the “50” in TICD50 should be subscripted.

**Response:** Thank you for pointing out this formatting issue. We have corrected the notation so that the “50” in TICD₅₀ now appears as a subscript in the revised supplementary figure (Fig. S4).

**Figure resubmission**

Following the journal’s recommendation, all figures in the revised submission have been processed using the PLOS NAAS tool. We have confirmed that the processed figures meet the required standards for print size and resolution, and we have uploaded the NAAS-processed files with this revision.

Once again, we thank the Editors and Reviewers for your time and constructive feedback. We hope that the revisions made in response to your comments have addressed all concerns and that the manuscript is now suitable for publication in PLOS Neglected Tropical Diseases.
